# Hepatic transcriptome and DNA methylation patterns following perinatal and chronic BPS exposure in male mice

**DOI:** 10.1186/s12864-020-07294-3

**Published:** 2020-12-09

**Authors:** Axelle Brulport, Daniel Vaiman, Elias Bou-Maroun, Marie-Christine Chagnon, Ludovic Le Corre

**Affiliations:** 1 Université de Bourgogne Franche-Comté, LNC UMR1231, F-21000 Dijon, France; 2 AgroSup, LNC UMR1231, 1 Esplanade Erasme, 21000 Dijon, France; 3 Nutrition Physiology and Toxicology Team (NUTox), INSERM, LNC UMR1231, F-21000 Dijon, France; 4 From Gametes to Birth Team (FGTB), INSERM, U1016, Institut Cochin, F-75014 Paris, France; 5 CNRS UMR8104, F-75014 Paris, France; 6 Université Sorbonne Paris Cité, F-75014 Paris, France; 7 Université de Bourgogne Franche-Comté, AgroSup Dijon, PAM UMR A 02.102, Procédés Alimentaires et Microbiologiques, F-21000 Dijon, France

**Keywords:** Bisphenol S, Perinatal and chronic exposure, Liver, DNA methylation, Transcriptome

## Abstract

**Background:**

Bisphenol S (BPS) is a common bisphenol A (BPA) substitute, since BPA is virtually banned worldwide. However, BPS and BPA have both endocrine disrupting properties. Their effects appear mostly in adulthood following perinatal exposures. The objective of the present study was to investigate the impact of perinatal and chronic exposure to BPS at the low dose of 1.5 μg/kg body weight/day on the transcriptome and methylome of the liver in 23 weeks-old C57BL6/J male mice.

**Results:**

This multi-omic study highlights a major impact of BPS on gene expression (374 significant deregulated genes) and Gene Set Enrichment Analysis show an enrichment focused on several biological pathways related to metabolic liver regulation. BPS exposure also induces a hypomethylation in 58.5% of the differentially methylated regions (DMR). Systematic connections were not found between gene expression and methylation profile excepted for 18 genes, including 4 genes involved in lipid metabolism pathways (Fasn, Hmgcr, Elovl6, Lpin1), which were downregulated and featured differentially methylated CpGs in their exons or introns.

**Conclusions:**

This descriptive study shows an impact of BPS on biological pathways mainly related to an integrative disruption of metabolism (energy metabolism, detoxification, protein and steroid metabolism) and, like most high-throughput studies, contributes to the identification of potential exposure biomarkers.

**Graphical abstract:**

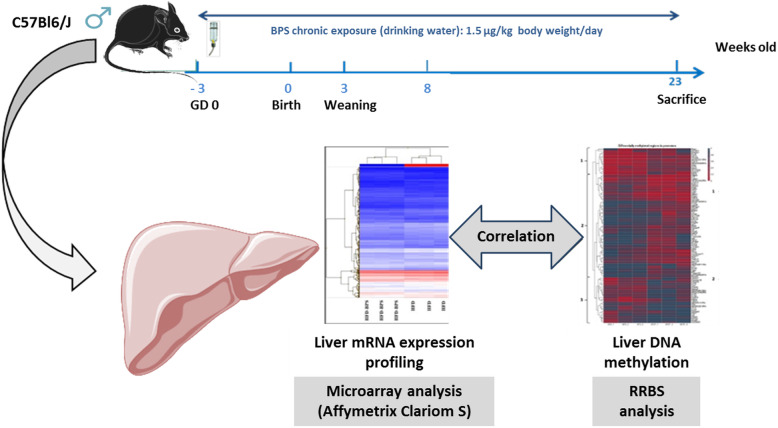

**Supplementary Information:**

The online version contains supplementary material available at 10.1186/s12864-020-07294-3.

## Background

Since the ban of Bisphenol A (BPA) in the baby bottles (Europe Union: regulation 321/2011, North America since 2009–2010, China since 2011) and the other use restrictions of this substance in relation to these reprotoxic and endocrine disruptor properties, manufacturers may use Bisphenol S (BPS), a structural analogue with similar endocrine disrupting and technological properties [1–3]. BPA and BPS are chemicals used as monomers in the manufacture of plastics and resins. Nearly sixty industrial sectors are potential users of these substances leading to daily human exposure. For example, they are present in polycarbonate (BPA) and polyether sulfone (BPS) plastics, paints, varnishes, thermal papers and paperboards, inks, glues, electronic and electrical components, cosmetics, medical, surgical and dental equipment, kitchen utensils and packaging in contact with food products [4]. Currently, BPS is approved for food contact materials with one restriction (EU 10/2011): a specific migration limit (SML) of 50 μg/kg. Food is the main route of BPS exposure [1]. BPS has been detected in many food products at doses ranging from 0.4 ng/ml in dairy products (United States), to 2.16 ng/g in meat (China) and 36.1 ng/g in carrots and peas (Spain) [4]. Furthermore, BPS is ubiquitously present in the environment (indoor air, surface water, sediment, sewage sludge, paper, food) and appears more resistant to environmental degradation than BPA and with a better level of dermal penetration [4–6]. In humans, BPS has been detected in urine (from 0.03 ng/ml in Korea to 0.4 ng/ml in the United States), blood (about 0.7 ng/ml in China), and in breast milk (0.23 μg/kg in France) [4, 7]. Interestingly, the BPS detection in U.S. adult urine samples increased from 25 to 74% between 2000 and 2014, in parallel to the decrease of BPA levels from 97 to 74% [8]. BPS daily intakes have been estimated at 0.023 μg/day for Korean, 0.316 μg/day for Americans and 1.67 μg/day for Japanese [9]. A recent physiologically based pharmacokinetic (PBPK) model shows that BPS exposure led to the highest internal concentrations of unconjugated bisphenol comparatively to other BPA substitutes [10]. Then, BPS toxicological impact might be more critical than those of BPA. Several studies describe a range of adverse effects that BPS can have on rodents including reprotoxicity [11–18], metabolic syndrome and obesity [19–23], hepatotoxicity [24, 25], cardiovascular toxicity [26, 27] and neurotoxicity [14, 28–30].

The liver is the key organ for metabolic homeostasis by ensuring the synthesis of most blood proteins, hormone biosynthesis and turnover, protein and bile synthesis, drug and energy metabolism [31]. Many endocrine disruptor compounds (EDCs) are sequestered and metabolized in the liver [31]. Several potential mechanisms by which EDC exposure might contribute to the pathogenesis of liver disease, including modulation of nuclear hormone receptor function and alteration of the epigenome, have been highlighted [31]. Epigenetics is the heritable alterations that regulate gene expression without change in DNA sequence. In mammals, the importance of epigenetics in environmental responses is increasingly studied in the field of metabolism [32]. DNA methylation of CpG islands, the first identified molecular mechanism of epigenetic regulation of gene expression, is associated with stable long-term changes in gene expression [33]. Although few direct links between environmental pollution, metabolic disturbances and epigenetic components have been demonstrated, associations between exposure to EDCs and changes in hepatic DNA methylation have been shown [32]. Perinatal and/or chronic exposures to environmental doses of BPA are associated with changes in liver DNA methylation and expression of genes involved in energy metabolism (Carnitine palmitoyltransferase: *Cpt1a*, Sterol regulatory-element binding protein-1c: *Srebp1c*, Fatty acid synthase: *Fas*, Glucokinase: *Gck*, Nuclear factor E2-related factor 2: *Nrf2* and Obesity-associated mesoderm-specific transcript: MEST) in rodents and humans [34–38].

Concerning BPS, there are few data available about its effects on the liver notably at molecular and epigenetic levels. Thus, the study aim was to analyzed transcriptome and methylome (DNA methylation) changes in the liver by performing a perinatal and chronic exposure of C57Bl6/J male mice during 26 weeks at 1.5 μg/kg b.w./day.

## Results

The list of abbreviations (names of genes) is available in the Additional file 1. Unprocessed data of mRNA differential gene expression are reported in Additional file 10.

### Expression profiling of liver genes after BPS perinatal and chronic exposure in 23 weeks old male mice

**Fig. 1:**
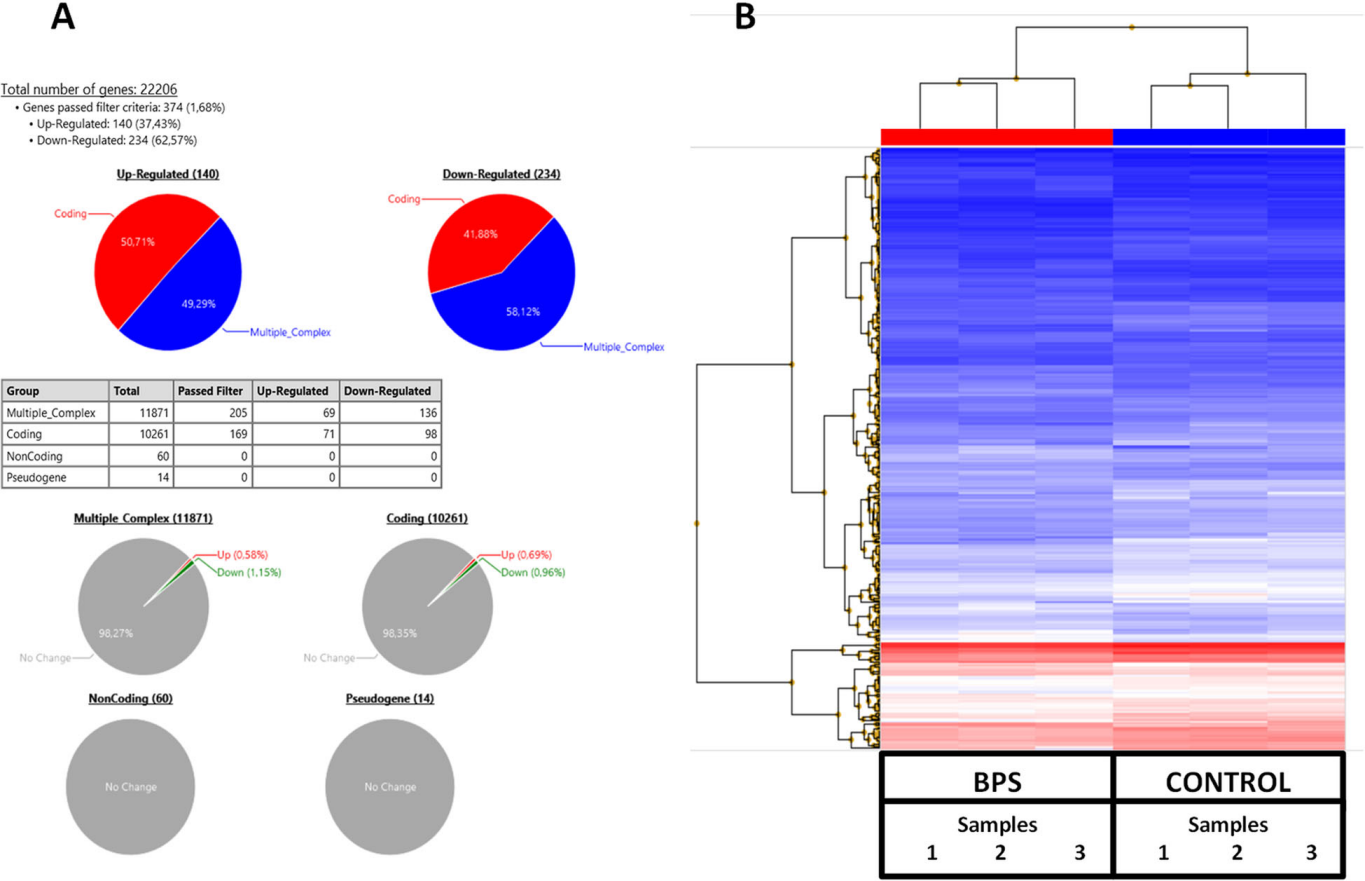
Expression profiling of liver up-regulated or down-regulated genes according to their category (**a**) and heatmap of differently expressed genes (**b**) when liver mRNA of C57Bl/6 J male mice exposed to BPS from GD0 to 23 weeks-old at 1.5 μg/kg b.w./day (BPS) is compared with liver mRNA of control mice by microarray. *n* = 3 pools of 3 animals each and by group. (Panel B color legend: i) at the top of the heatmap, a red band for SD BPS 1.5 and a blue band for SD; ii) in the heatmap, a color scale configuration with a gradient from dark blue for a minimum signal of 2.5, an average signal of 10.75 in white and a maximum signal of 19 in dark red)

Among the 22,206 genes studied in the microarray experiment, using a threshold of 1.5-fold and a *p*-value < 0.05, 374 genes were found deregulated (140 up-regulated and 234 down-regulated) which represents 1.7% of the total genes investigated (Fig. 1a). All deregulated genes belong to the categories Coding and Multiple_Complex (gene containing more than one locus type, such as a gene encompassing a miRNA in an intron, or a complex gene family) which are the mainly interrogated by this transcriptomic analysis (Fig. 1a). Concerning up-regulated genes, 50.7% are coding genes (i.e. 0.7% of the genes studied for this category) and 49.3% belong to the Multiple_Complex group (i.e. 0.6% of the genes included in this group) (Fig. 1a). For down-regulated genes, the coding genes and the Multiple_Complex group represents 41.9% (i.e. 1% of the category) and 58.12% (i.e. 1.2% of the group), respectively (Fig. 1a).

From the 374 differentially expressed genes, a semi-supervised analysis unambiguously segregates the animals according to BPS exposure (Fig. 1B; genes in the heatmap are listed in Additional file 8).

### Gene set enrichment analysis highlights the alterations of gene pathways under BPS treatment

**Fig. 2:**
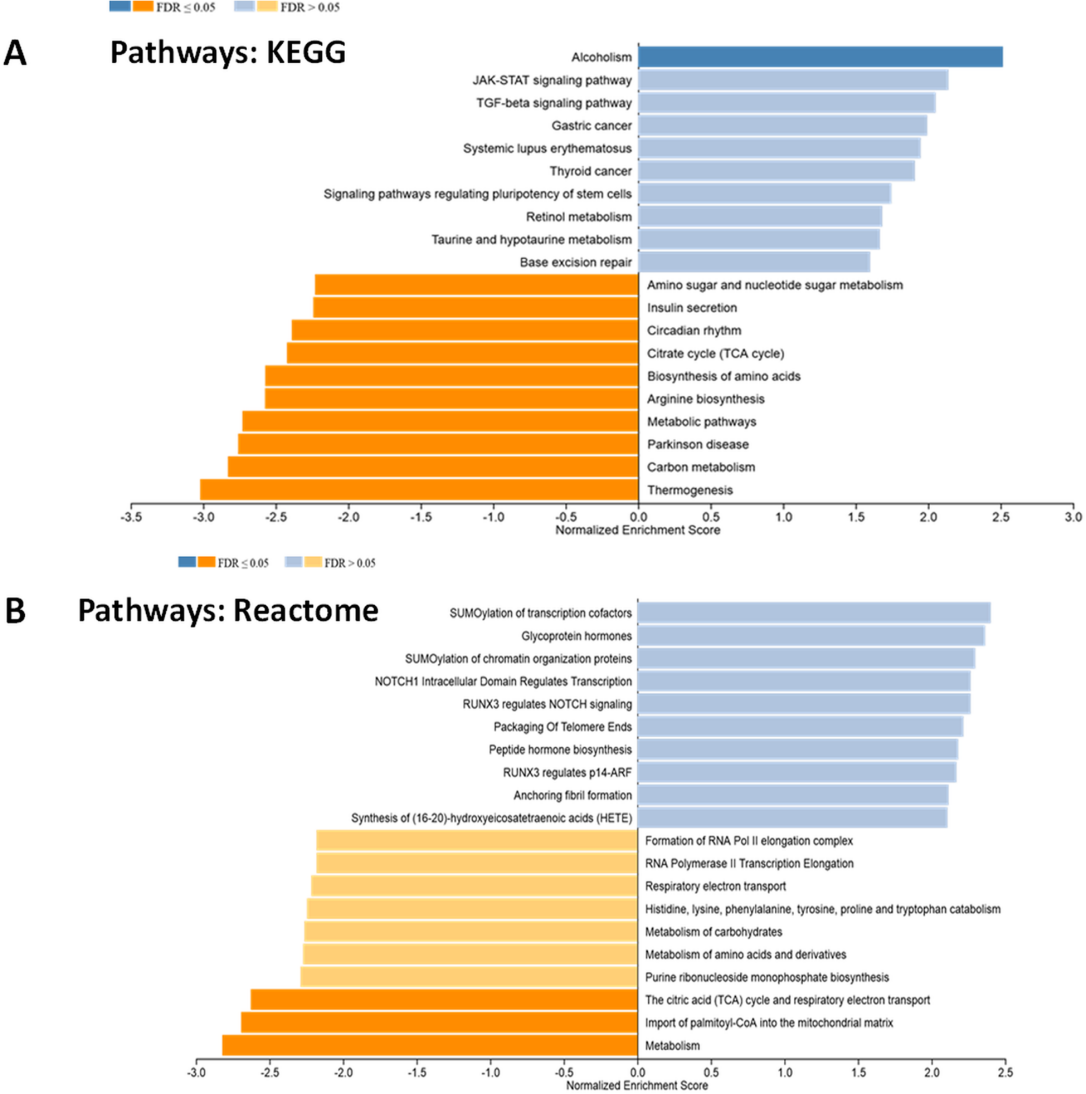
Functional Gene Set Enrichment Analysis identified by the databases of pathways KEGG (**a**) or Reactome (**b**) for down-regulated genes and overexpressed genes using the total gene set when liver mRNA of C57Bl/6 J male mice exposed to BPS from GD0 to 23 weeks-old at 1.5 μg/kg b.w./day is compared with liver mRNA of control mice by microarray. *n* = 3 pools of 3 animals each and by group

With WebGestalt, a WEB-based gene set analysis tool kit, we performed a Gene Set Enrichment Analysis using the total gene set (22,206 genes), in comparison with two pathway databases, KEGG and Reactome [39, 40]. In the liver of male mice exposed to BPS: alcoholism, thermogenesis, carbon metabolism, Parkinson disease, metabolic pathways, arginine biosynthesis, biosynthesis of amino acids, citrate cycle, circadian rhythm, insulin secretion and amino sugar and nucleotide sugar metabolism pathways were all significantly enriched according to KEGG database (Fig. 2a). Only alcoholism pathway presents a significant positive Normalized Enrichment Score (NES) (Fig. 2a) (Additional file 4).

The same type of analyses by GSEA carried out with reference to the Reactome database revealed only significant negative NES for metabolism and mitochondrial function pathways (Fig. 2b) (Additional file 4), with the TCA found consistently in Reactome and KEGG pathway repositories.

When the analyses were focused exclusively on the 374 genes which passed the filter criteria (1.5-fold and *p*-value < 0.05), no significant enrichment has been identified regardless of the database used, suggesting that the gene ontology clustering results rather from several genes that are moderately deregulated, inside relevant cascades, and not a limited number of genes strongly deregulated (Additional file 2).

In addition to the GSEA approaches on KEGG and Reactome pathways, we analyzed the dataset for enrichment in genes presenting specific transcription factor binding sites. We found normalized enrichment scores –NES- > 2 for CREBP1_01, TTAYRTAA_E4BP4_01, CEBP_Q3, AHRARNT_02, which are found in the promoters of target genes involved in inflammation, detoxification, regulation of circadian rhythms, energy metabolism and tumorigenesis. They present with an integrated deregulation of liver functions in mice exposed to BPS which may compromise later the healthy aging of animals (additional files 3 and 5).

### Connections between significantly deregulated genes and those involved in pathways

**Fig. 3:**
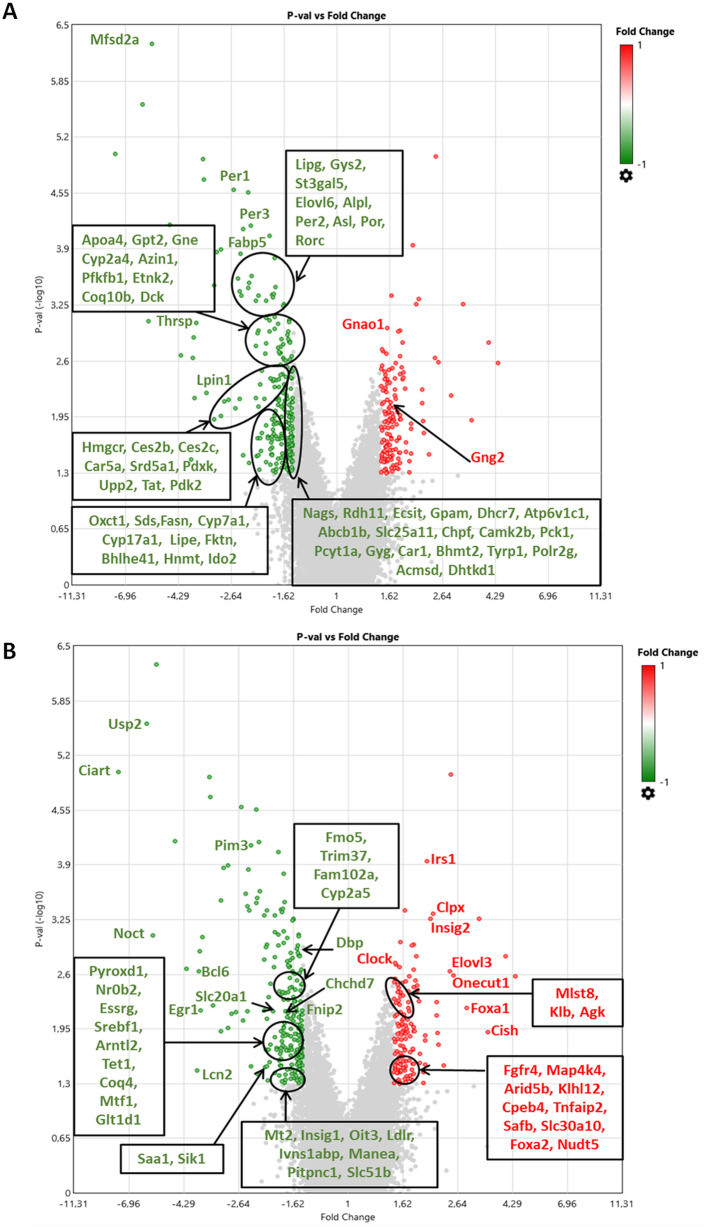
Expression profiling of liver up-regulated (left side) or down-regulated (right side) genes according to their level of deregulation in volcano plot for genes passed filter criteria (Fold Change ≤ − 1.5 or ≥ 1.5 and *p*-value < 0.05) and associated to Functional Gene Set Enrichment (**a**) or to liver physiopathology without being included in pathways (**b**) when liver mRNA of C57Bl/6 J male mice exposed to BPS from GD0 to 23 weeks-old at 1.5 μg/kg b.w./day is compared with liver mRNA of control mice by microarray. *n* = 3 pools of 3 animals each and by group

Most of the significantly deregulated genes found in significant enriched pathways are involved in metabolic process, mainly in glucose and fat metabolism (Lipg, Gys2, Elovl6, Rorc, Per2, Mfsd2a, Fabp5, Lipe, Pcyt1a, Thrsp, Lpin, Fasn, Apoa4, Cyp2a4, Cyp7a1, Pfkfb1, Pck1, Gyg, Fktn, Etnk2, Hmgcr, Ces2b, Ces2c, Car5a, Dhcr7) and to a lesser extent in amino acids and glycoaminoglycan metabolism (Chpf, Gpt2, Tat, Sds, Hnmt, Ido2, Bhmt2, Tyrp1, Acmsd, Dhtkd1), xenobiotic metabolism (Por, Cyp2a4, Cyp7a1, Ces2b, Ces2c, Abcb1b, Rdh11), urea cycle (Car1, Nags, Asl, Car5a), steroid metabolism (Srd5a1, Por, Cyp2a4, Cyp7a1, Cyp17a1), nucleotide metabolism (Dck, Upp2) and vitamin metabolism (Rdh11, Pdxk) (Fig. 3a) (Additional file 4).

The other genes identified are involved in mitochondrial function (Coq10b, Pdk2, Oxct1, Bhlhe41, Ecsit, Gpam, Slc25a11, Pck1), circadian rhythm (Per1, Per2, Per3, Rorc), nervous transmission (Gnao1, Atp6v1c1, Camk2b, Acmsd), transcription regulation (Polr2g), bone mineralization (Alpl) and intracellular polyamines regulation (Azin1) (Fig. 3a) (Additional file 6). It is important to note that all these genes are down-regulated by the BPS treatment. Only genes related to transmembrane signaling are either up (Gnao1, Gng2) or down-regulated (St3gal5, Gne) (Fig. 3a) (Additional file 4).

### Other significantly deregulated genes involved in liver physiopathology but not included in pathways

Some genes have similar biological functions than those identified by enrichment analyses, with the difference that they are either up- or down-regulated. We found genes associated with pathways of circadian rhythm (Usp2, Ciart, Noct, Slc20a1, Egr1, Dbp, Arntl2, Clock), mitochondrial function (Chchd7, Coq4, Clpx, Slc30a10, Agk), transcription regulation (Trim37, Tet1, Foxa1, Foxa2, Arid5b, Safb, Nudt5, Ivns1abp) and metabolism (Fnip2, Insig1, Ldlr, Saa1, Srebf1, Mtf1, Irs1, Insig2, Elovl3, Onecut1, Foxa1, Foxa2, Fgfr4, Pitpnc1 for energy metabolism, Fmo5 and Cyp2a5 for xenobiotic and steroid metabolism, Manea for protein metabolism and Nudt5 for nucleotide metabolism) (Fig. 3b).

New biological processes such as tumorigenesis and cell cycle control (Pim3, Bcl6, Egr1, Oit3, Onecut1, Foxa1, Foxa2, Mlst8, Fgfr4, Glt1d1), inflammation (Lcn2, Bcl6, Egr1, Saa1, Cish, Map 4 k4, Tnfaip2, Il6ra) and cellular stress (Mtf1, Mt2, Pyroxd1, Cpeb4, Slc30a10) have emerged (Fig. 3b). Interestingly, the genes involved in bile acids metabolism (Klb, Fgfr4, Klhl12, Tnfaip2, Slc51b) are mainly up-regulated and those implicated in estrogen/androgen action (Fam102a, Nr0b2, Esrrg) are only down-regulated (Fig. 3b).

### Untargeted assessment of genome-wide alterations in liver DNA methylation by reduced representation bisulfite sequencing (RRBS) under BPS treatment

**Fig. 4:**
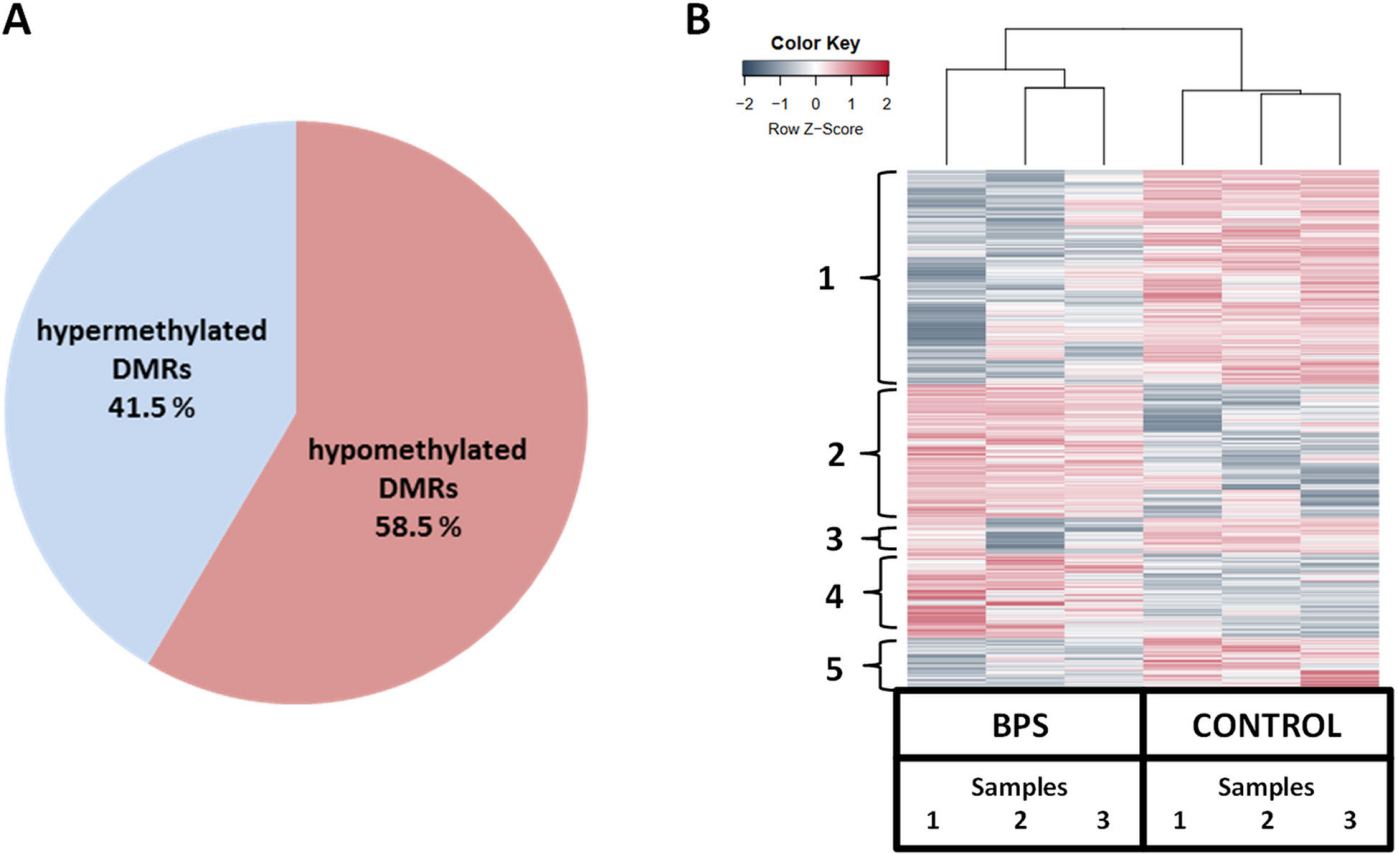
Percentage of hyper and hypomethylated regions with a percent methylation difference cutoff of 25% and q-value of 0.01(**a**), and heatmap of Differentially Methylated Regions (DMRs) (width of 1000 nucleotides) (**b**) when liver DNA of C57Bl/6 J male mice exposed to BPS from GD0 to 23 weeks-old at 1.5 μg/kg b.w./day (BPS) is compared with liver DNA of control mice by RRBS. *n* = 3 pools of 3 animals each and by group

To investigate whether changes in DNA methylation in the liver were induced by BPS, we analyzed the liver DNA by RRBS which allows to analyze 70% of CpGs islands encompassed in ≈1% of the genome in mice. Concerning the analysis at the Differentially Methylated Regions (DMRs) level, with a percent methylation difference cutoff of 25% and q-value of 0.01, this BPS perinatal and chronic exposure was associated with a significant hypo or hyper methylation of 1811 DMRs (width of 1000 nucleotides per DMR), which corresponds to 7.3% of DMRs assessed by RRBS approach, in 23 week-old male mice fed a standard diet. Among this DMRs, the percentage of hypermethylated regions was 41.5% against 58.5% for hypomethylated regions (Fig. 4a). These data are in agreement with the analyses performed at the CpG dinucleotide level (44% of hypermethylated CpGs and 56% of hypomethylated CpGs).

Semi-supervised hierarchical classification revealed five clusters of DMR according to the BPS exposure (Fig. 4b). The 1, 3, and 5 clusters contain regions down-methylated by the treatment. In contrast, the clusters 2 and 4 contain regions up-methylated in this context (Fig. 4b).

### Genomic distribution of differentially methylated CpGs and DMRs

In the current experiment, between 343 and 605 million of cytosine residues were analyzed per sample. The methylated/non-methylated cytosine ratios are in the same range 41.5% for control mice and 42% for BPS exposed mice (Additional file 7). Among the cytosines analyzed, 20.7% are located inside CpGs islands, 22.5% are in a CpHpG context and 56.8% are in a CpHpH context (H = A, C or T). This is consistent in all samples and with results typically obtained in an RRBS analysis with a mouse sample. Even if the cytosines analyzed in the CpG islands represent only 20.7% of those analyzed, more than 40% are methylated, while only 0.5 to 0.4% of the cytosines in a CpHpG or CpHpH context are methylated, respectively (Additional file 7).

**Fig. 5:**
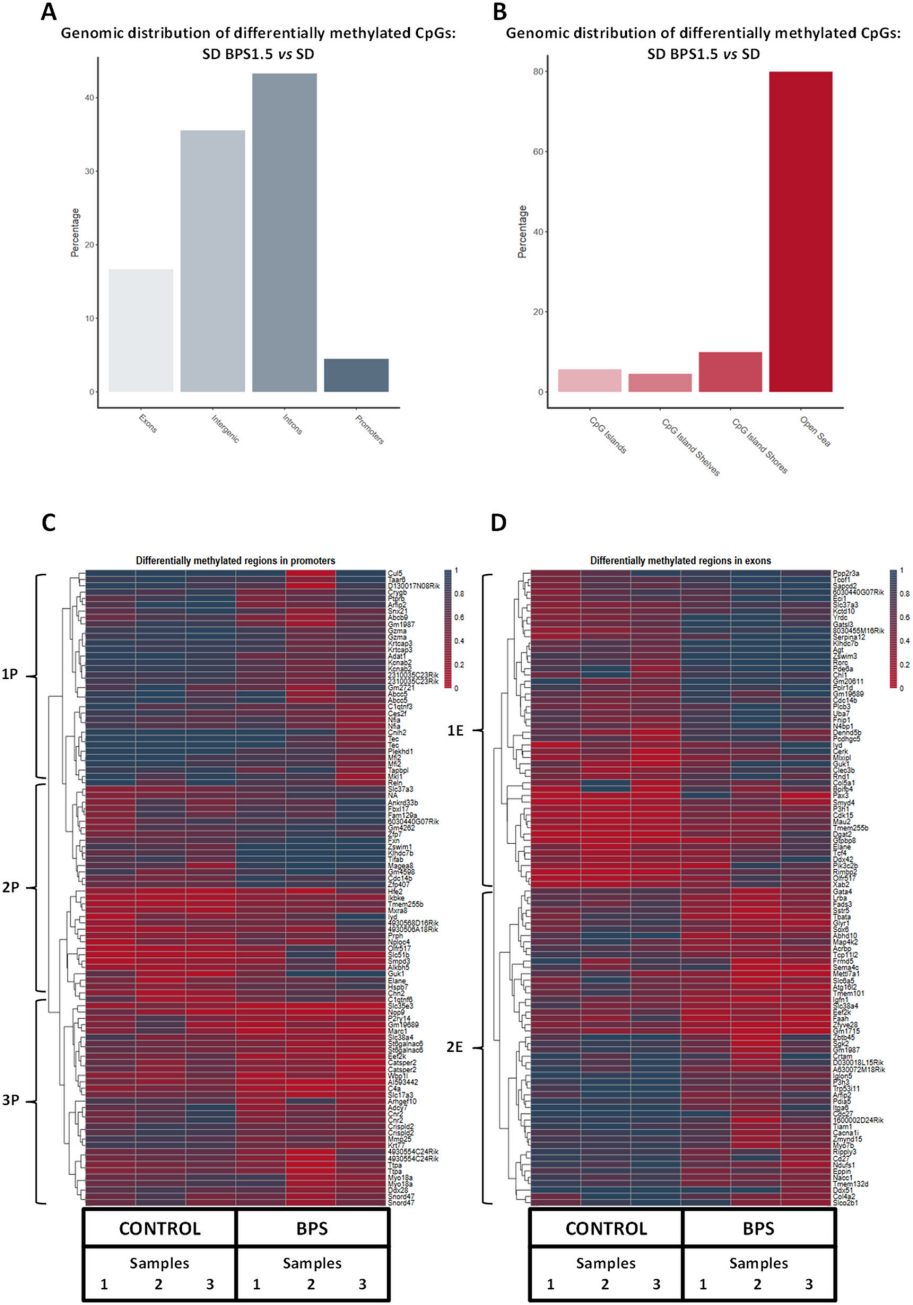
The differentially methylated CpGs (DMCs) annotated with different genomic regions (**a**), as well as CpG island and shore coordinates (**b**) and their distribution. Heatmaps of top 100 Differentially Methylated Regions (DMRs) (width of 1000 nucleotides) located in promoters (**c**) or in exons (**d**). The data were from a RRBS analysis of the comparison between liver DNA of C57Bl/6 J male mice exposed to BPS from GD0 to 23 weeks-old at 1.5 μg/kg b.w./day and control mice. *n* = 3 pools of 3 animals each and by group

For the BPS vs CONTROL comparison, 5.7% of differentially methylated CpGs were found located within CpG islands (defined by the following criteria: > 200 bp length, GC percentage > 50% and observed/expected CpG ratio > 60%), 4.6% within CpG shelves (2–4 kb from CpG island), 9.9% within CpG shores (regions up to 2 kb away from CpG islands) and 79.9% in open sea (isolated CpGs in the genome) [41] (Fig. 5b) (Additional file 7). Concerning the gene localization of the alterations, we identified 16.8% of differentially methylated CpGs located in exons, 34.2% in intergenic regions, 44.6% in introns and 4.4% in promoter regions (Fig. 5a). For DMRs, 47.8% are in intergenic regions, 44.2% in introns, 5.9% in exons and 2.1% in promoter regions (Additional file 7).

### Identification and clustering of genes with DMRs located in promoter or exons areas

A semi-supervised analysis of the 100 DMRs most affected by BPS exposure, allowed us to identify three clusters for DMRs located in promoters (Fig. 5c, Additional file 11). Clusters 1P and 3P correspond to genes in hypo-methylated DMRs and cluster 2P to those in hyper-methylated DMRs (Fig. 5c). Concerning exons, DMRs clustering has highlighted one hyper-methylated (cluster 1E) and one hypo-methylated (cluster 2E) cluster (Fig. 5d, Additional file 11). The GSEA approaches on KEGG and Reactome pathways shown there is no significant pathway that emerges (FDR > 0.05) either by analyzing DMC in exons and / or promoters (Additional file 9).

### Connections between DNA methylation and gene expression alterations

**Fig. 6:**
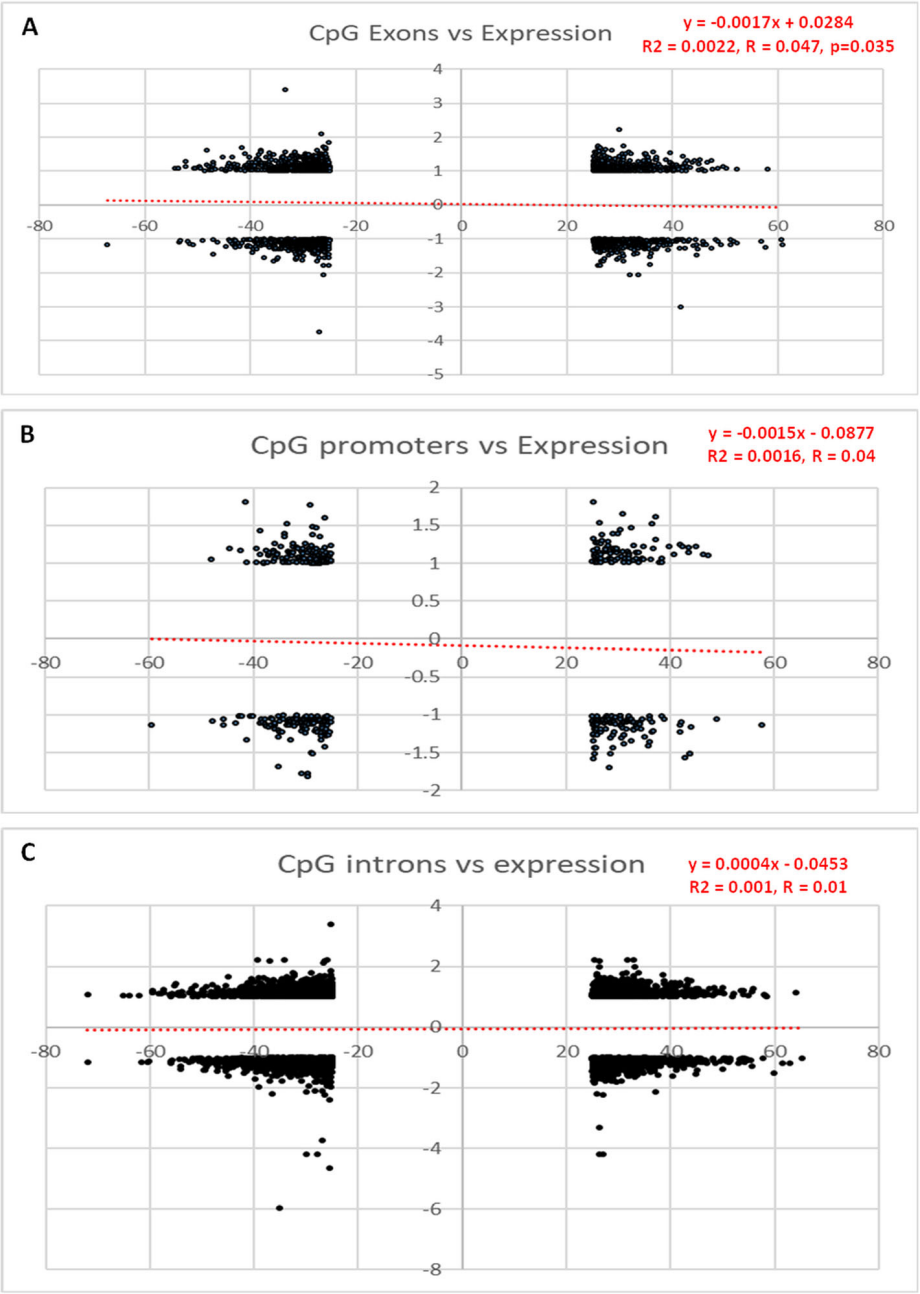
Linear regression between liver DNA methylation and mRNA expression levels in exons (**a**), promoters (**b**) and introns (**c**) of C57Bl/6 J male mice exposed or not to BPS from GD0 to 23 weeks-old at 1.5 μg/kg b.w./day. *n* = 3 pools of 3 animals each and by group

To study the connections between DNA methylation and gene expression, all the CpGs located in a gene sequence and that were found significantly differently methylated were investigated. The expression profiles of the genes were monitored among the 22,206 genes studied in the microarray experiment. Then, linear regressions were carried out between methylation and expression levels (Fig. 6). Concerning CpGs located in exons, there is a weak but significant negative correlation between methylation and expression for 2016 genes (Fig. 6a). No significant correlations were found for promoters (among 442 genes: Fig. 6b) and introns (among 5316 genes: Fig. 6c). These considerations do not mean that genes involved in a given cascade are not modified at the transcription level; for instance, a ‘bandmaster’ gene could indeed be regulated through methylation changes, and activate or inhibit many genes whose methylation profile is not altered; besides, other epigenetic modes of regulation than DNA methylation (miRNA) could drive transgenerational programming and trigger modifications of expression.

By focusing the analysis of these connections only to the 374 genes significantly deregulated, 18 genes showed hypo- or hypermethylated CpGs on their exons or introns (Additional file 6) and 10 genes in their promotors. No relationship between CpGs location, methylation status and the up- or downregulation of gene expression was observed (Additional file 6). These genes were involved in different biological process (metabolism, circadian rhythms, spermatogenesis, immune response, bone mineralization, vascular growth, prostate development) and ubiquitous cellular process (G-protein signaling, mitochondrial function, transcription regulation, potassium channel activity) (Additional file 6).

**Fig. 7:**
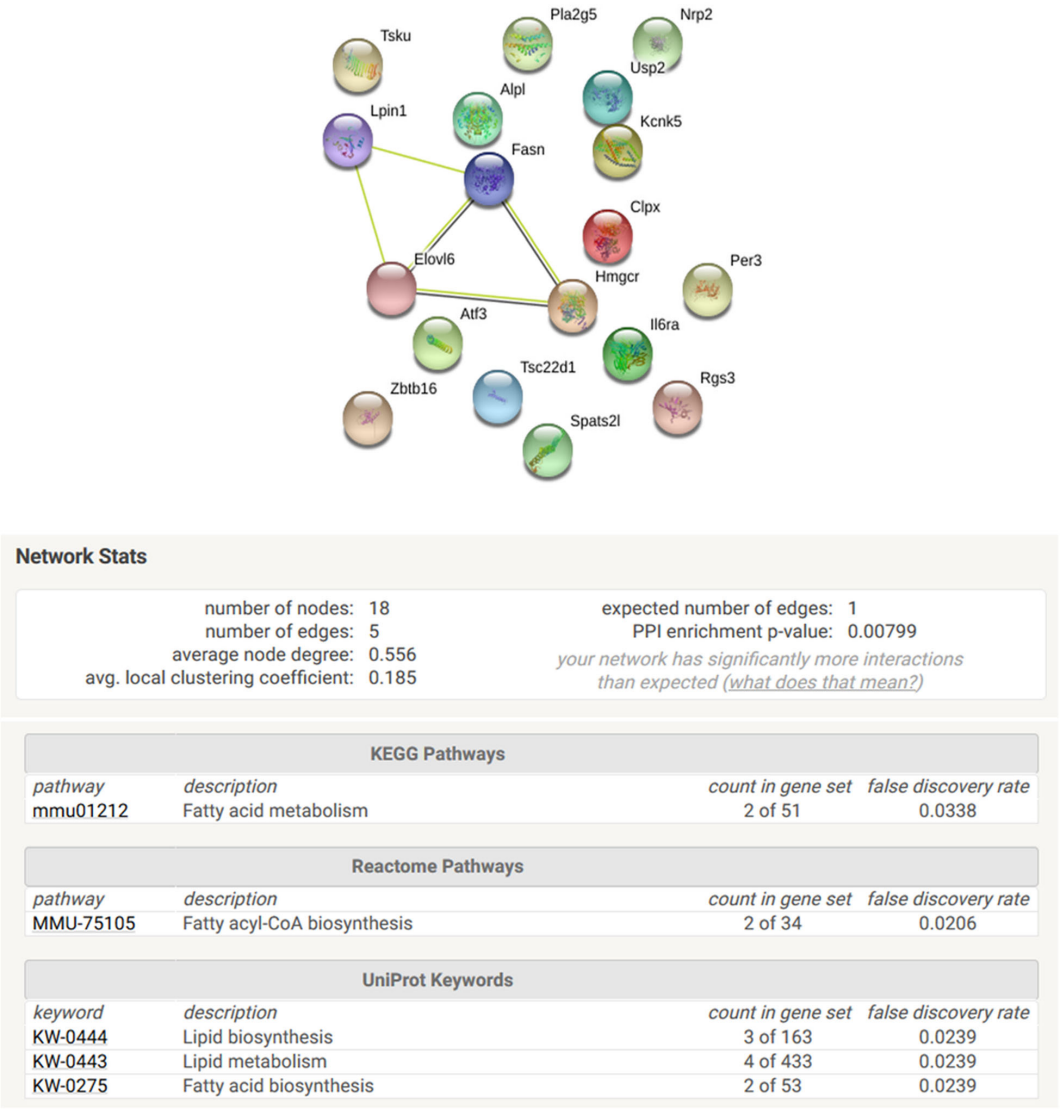
Functional Gene Set Enrichment Analysis (with String database v 11.0 (https://string-db.org/)) for genes with correlation between significant DNA hypo- or hypermethylation and mRNA up- or downregulation when liver DNA and mRNA of C57Bl/6 J male mice exposed to BPS from GD0 to 23 weeks-old at 1.5 μg/kg b.w./day are compared with liver DNA and mRNA of control mice. n = 3 pools of 3 animals each and by group

Interestingly, Functional Gene Set Enrichment analysis highlighted a significant enrichment in pathways related to fatty acid metabolism involving the genes Fasn, Lpin1, Elovl6 and Hmgcr (Fig. 7).

## Discussion

In this study, we highlight the impacts of a BPS perinatal and chronic exposure on the transcriptome and the DNA methylation pattern in the liver of male mice. Here, only male mice were investigated. Indeed, the present study echoes back to our previous article where it was shown that the same BPS exposure only induced overweight in male mice fed to high fat diet [19] and was associated with alterations in liver DNA methylation and transcriptomic profiles [42]. Also, the liver is the most sexually differentiated organ in mammals with a major impact on drug and steroid hormone (no aromatase expression in male mouse liver, for example) and energy metabolism (more efficient lipid metabolism in female mice). As BPS exhibits estrogenic and metabolic disruptor properties, male mice were used to limit sex-specific physiological effects [43].

The BPS exposure data of the European population are still scared, we conducted this study at the dose of 1.5 μg/kg b.w./day, which corresponded to the BPA exposure of human adults consuming 3 kg of commercial products daily in 2015 [44]. Since 2010, BPS being used as a substitute for BPA, it is very probably that human exposure to BPS will be of this range in the next years. Moreover, even if the substitution of BPA can only be partial depending on the regulations of the countries, BPS exhibits a higher body burden and bioavailability than BPA [5].

In relation to the study aim, we performed bioinformatics analysis to determine i) the biological pathways potentially involved - based on differential mRNA expression - ii) genes with differentially methylated cytosine and iii) the connection between mRNA expression and methylation status of genes. Wild-type transcriptomic and genomic methylation profiles were generated from pools of DNA and RNA from three mouse liver samples. Omic analyzes require high quality samples. As a result, the DNA and RNA pools were not composed of the same liver samples. These non-homogeneous pools did not allow a more in-depth analysis linking RNA expression and DNA methylation and can hide inter-individual heterogeneity. However, the current results have highlighted biological pathways or target genes impacted following BPS perinatal and chronic exposure.

All the biological pathways identified through biostatistics gene ontology enrichment analyses, revealed an impact of BPS on gene expression of the major liver functions: energy metabolism (ketone bodies, glucose and fat metabolism), detoxification metabolism (in part highlighted by the enrichment of the alcoholism pathway), bile acids metabolism and synthesis and proteins and hormone biosynthesis and turnover. The genes associated with these biological pathways refer to pathophysiological mechanisms involving alteration of mitochondrial functions (related to oxidative stress increasing), development of inflammatory processes, disruption of circadian rhythms, impairment of intra and extracellular communication (particularly exocytosis phenomena in this study) and an impact on epigenetic regulatory mechanisms (by impacting the epigenetic machinery and one-carbon metabolism for methylation events). Interestingly, in the significant Alcoholism enriched pathway, an important part of leading edge are genes involved in transcription regulation via post-translational modification of histones (Additional file 6). Energy metabolism is the pivotal point of the observed deregulations. Disruption in the circadian rhythm alters metabolic homeostasis (feeding-fasting cycle, glucose and lipid metabolism and thermogenesis among others) and is associated with metabolic syndrome and non-alcoholic fatty liver disease (NAFLD) [45]. Several multiomics studies to characterize the pathophysiology of NAFLD in rodents have highlighted the role of mitochondrial dysfunction in this pathology [46–48]. Moreover, oxidative stress, in addition to induced cell damage and apoptosis generates a release of proinflammatory cytokines resulting in hepatic inflammation, fibrosis and cirrhosis, three histological signatures of the NAFLD [49, 50]. In accordance with these data, Zhang et al. (2018) reported that subchronic Bisphenol S exposure affects liver function in mice involving oxidative stress [25].

Bile acids regulated energy metabolism and immunity and their homeostasis disruption results in pathological cholestasis and in metabolic liver diseases [51]. To our knowledge, there is no study on the impact of BPS on detoxification mechanisms, but modifications of these processes could alter the functioning of the brain-liver axis. Liver plays a critical role by providing vital nutrients to the brain and by detoxifying the splanchnic blood. Impairment of liver detoxification function can lead to increased neurotoxins and altered brain metabolism promoting inflammation and the development of neurological pathology [52]. These metabolic alterations may also be related to neurodegenerative disorder like Alzheimer’s or Parkinson’s disease [53, 54]. Furthermore, a decrease of cytochrome P450 activity can affect drug and endogen metabolism and increasing susceptibility to drug-induced liver injury and hepatocellular carcinoma [55, 56].

In this study, few genes among those that are significantly deregulated show a change in their methylation profile on associated CpGs. Interestingly, for genes which connections were found between mRNA expression and DNA methylation changes, only the enrichment of pathways related to fatty acid metabolism was significant. This is consistent with the role of BPS as a metabolic disruptor [19]. Given the essential role of lipids in physiology, it seems consistent to hypothesize that it is potentially by disrupting lipid metabolism that BPS is involved in different physiopathological processes.

Although correlations between methylation and gene expression are rare, in this study BPS has a major impact on the hepatic methylome. In most cases, these epigenetic marks are located in non-coding regions, but this does not mean that they do not have a potential impact on transcription. In non-coding regions, the hypomethylation of repeated sequences can lead to chromosomal instability and promoting tumorigenesis processes (intergenic regions) or induce the “unmasking” of regions regulating the expression of the adjacent gene (introns) [57–60]. For coding regions, the hypermethylation of an exon can be associated with both activation and repression of transcription [61–63]. For promoters, hypomethylation in the TSS or in the insulator region would promote transcription [64–67]. The role of methylation status at enhances/repressors sites is more disputable [68]. Moreover, DNA methylation is not the only epigenetic mark that influences transcription. It interacts with the post-transcriptional modification of histones and the epigenetic modulators that are microRNAs to participate in compression or relaxation processes of chromatin structures that contribute to gene expression regulation [69, 70]. Few studies have shown a causal link between environmental factors and epigenetic changes. However, many correlations between developmental exposures of endocrine disruptors and changes in gene expression are associated with DNA methylation, variations in the histone code pattern or interference with microRNAs [71]. Global DNA hypomethylation highlighted in this paper may potentially be related to the combination of a weak and non-significant decrease of the expression levels of DNA methyltransferases involved in de novo methylation (Dnmt1, Dnmt3a, Dnmt3b) with all of these other epigenetic regulations. The overall hypomethylation of DNA induced by BPS exposure and chromatin status influences the effect of some transcription factors on their binding site [72]. Interestingly, in animals exposed to BPS, the only transcription factor with significant enrichment score interacts with many genes encoding histone proteins. It should be noted that this is also the case for many other transcription factors with a positive NES score (Additional file 5). Several other physiological hypotheses may also explain this lack of correlation between methylation level and gene expression. The liver is an organ composed of several cell types with distinct functions. The liver is composed of 80% parenchymal cells (hepatocytes) involved in many metabolic processes and 20% non-parenchymal liver cells (Kupffer cells, liver sinusoidal endothelial cells and hepatic stellate cells) implicated in immune regulation, intercellular trafficking, vitamin A storage and fibrosis [73]. These cells may therefore respond differently to BPS exposure. The fact that single cell studies were not conducted may contribute in part to these observations. Another point of discussion, more specifically to our experimental conditions and to the key role of liver in the regulation of metabolic homeostasis by adapting to nutritional status, the mice were fasting for 4 h at the time of sacrifice. Hypothetically, this duration allows changes in mRNA expression in the liver but not to modify the DNA methylation profile. Global hypomethylation events are enriched for repetitive sequences and thought to be responsible for the reactivation of retrotransposon elements during aging, as one potential mechanism leading to a higher incidence of cancer [74]. In our experimental context the presence of global DNA hypomethylation in young animals exposed to BPS (23-weeks old to sacrifice) may suggest that these animals will develop a characteristic phenotype during aging or in association with other risk factors such as high fat diet as we previously described [39].

## Conclusions

To our knowledge, this is the first time that the impact of perinatal and chronic exposure to BPS on the mouse transcriptome and DNA methylation pattern has been studied. We have demonstrated through two omic approaches (microarray and RRBS), that BPS impacts genes involved in hepatic metabolism. Like most high-throughput studies, this study contributes to the identification of potential exposure biomarkers.

## Methods

### Animals and materials

Pregnant C57Bl/6 J mice were acquired from Charles Rivers (L’Arbresle, France). Bisphenol S (BPS) was provided by Sigma-Aldrich (Saint Quentin Fallavier, France). The standard diet (SD) 4RF21 was purchased from Mucedola (Mucedola, Milano, Italy). This diet is certified as estrogen free and estrogenic activities were evaluated. The phytoestrogen level is certified below 4 ppb (parts per billion) according to international standards (U.S. Food and Drug Administration National Center for Toxicology Research Standard No. 2, September 5, 1973).

### Experimental strategy

Twenty-two pregnant mice were individually housed in a 12 h light-dark cycle at 22 °C in a conventional animal facility and allowed free access to food and water. From the first day of gestation (GD0), the pregnant C57Bl/6 J mice were divided into two groups and either exposed or not to BPS diluted in the drinking water. GD0 was defined from the detection of the vaginal plug. The treatment period was extended during lactation and in pups after weaning up to 23 weeks of age. At weaning, the male offspring were randomly separated into two groups as follows: fifteen in the group exposed to BPS and sixteen in the control group. In each cage was housed a maximum of five mice. After weaning, the litters were mixed randomly to minimize a possible litter effect. In a cage, no mouse came from the same dam. The suitable concentration of BPS was based on the estimated average daily water consumption in C57Bl/6 J mice (about 7 ml / 30 g mouse) to achieve a predicted BPS exposure of 1.5 μg / kg bw / day. Then, we checked the BPS exposure as follows: weekly water intake was evaluated by calculating the difference between the amount of water placed in the water bottle at the beginning and the amount remaining after 7 days. The levels of BPS consumed each week were normalized by day and the number of mice by cage. Thus, we obtained an average BPS intake of 1.13 (±0.084) μg/kg bw/day. The BPS was dissolved in absolute ethanol (final concentration 0.1% in drinking water). The control group were exposed to 0.1% ethanol in drinking water. Bottles and cages were made of polypropylene (bisphenol-free). At 23 weeks old, mice were fasting for 4 h before to be sacrificed in the early afternoon. The euthanasia and the dissections were organized in small sessions, mixing treated and control animals. Anesthesia of the mice was performed with isoflurane before their euthanasia by cervical dislocation. The livers were removed, weighed, frozen in liquid nitrogen and stored at − 80 °C.

### Transcriptomic analysis

#### RNA extraction and microarray analysis

Liver samples were crushed and homogenized using Lysing Matrix D™ tubes (MP Biomedical, Illkirch-Graffenstaden, France) and the tissue homogenizer Precellys™24 (Bertin technologies, Montigny-le-Bretonneux, France). For each mouse, liver sample was from the medial liver lobe. The extraction of total RNA were performed using Tri-reagent™ (Sigma-Aldrich, Saint Quentin Fallavier, France). RNA quality (RNA Integrity Number) and quantification were realized by capillary electrophoresis (Agilent bioanalyzer 2100, Les Ullis, France). By using cartridges of Affymetrix Clariom S array hybridization, isolated cRNA of three points per condition were analyzed for global gene expression. The standard validated protocol were performed at the Genomics Platform of the Cochin Institute. Each sample consisted of a pool of RNA prepared from the liver of three separate mice and different litters. The pools were formed according to the physiological characteristics of the mice (body and liver weights, fat mass, liver triglyceride content) and the nucleic acid quality, to have three homogeneous samples. For all samples, equal amounts of nucleic acid were used for each mouse. The genes showing a significant deregulation (p⩽0.05) were converted to ENSEMBL ID with Biomart and mm10 version of the mouse genome (http://www.ensembl.org/biomart).

#### mRNA gene expression signature and gene set enrichment analysis (GSEA)

Files type .CEL were generated by the Genomics Platform of the Cochin Institute. They were analyzed using the Transcription Analysis Console 4.0 (TAC 4.0) from Affymetrix (Applied Biosystems). The differential mRNA gene expression was represented in the form of pie chart and heatmap.

A Functional Gene Set Enrichment Analysis (GSEA), for down-regulated and overexpressed genes, was performed with WebGestalt 2019 (WEB-based Gene SeT AnaLysis Toolkit) (http://www.webgestalt.org/) by using two pathway databases: KEGG and Reactome.

### Analysis of genome-wide methylation profile

#### Extraction of liver DNA

The lysis of liver samples was performed as described in 5.3 section. The samples were digested overnight with 20 μl of proteinase K (20 mg/ml) and 9 μl of RNase A (20 mg/ml). Before the DNA extraction, samples were treated with 300 U of RNase T1, 30 min at 37 °C. Then, GenElute Mammalian Genomic DNA Miniprep kit™ (Sigma Aldrich, Saint Quentin Fallavier, France) were used following the manufacturer’s instructions. DNA concentration was measured using the Qubit® dsDNA BR Assay Kit (Thermo Fisher Scientific) and DNA quality was analyzed using the Fragment Analyzer™ and the DNF-487 Standard Sensitivity Genomic DNA Analysis Kit (Advanced Analytical).

The composition of DNA and RNA pools was different as they depended on the respective nucleic acid quality.

#### Genome-wide methylation profile by reduced representation bisulfite sequencing (RRBS)

RRBS, an untargeted method allowing the analysis of 70% of CpGs islands of the mouse genome, was executed by Diagenode’s DNA Methylation Profiling (RRBS Service) (Diagenode Cat# G02020000). RRBS analysis were performed as previously described [42]. Briefly, libraries have been prepared using Diagenode’s Premium RRBS Kit (Diagenode Cat# C02030033) and RRBS library pools were sequenced on a HiSeq3000 (Illumina) using 50 bp single-read sequencing. The sequenced read quality was assessed with FastQC, the cleaning step was performed using Trim Galore! version 0.4.5_dev and the alignment to the *Mus musculus* reference genome (Genome Reference Consortium m38 (mm10)) has been carried out using bismark v0.16.1 [75, 76]. The sequencing results which are an indicator of the quality of the analysis are summarized in Table 1. Differentially methylated CpGs (DMCs) and regions (DMRs; window and step size of 1000 bp) were defined by pairwise comparisons. DMCs and DMRs were considered statistically significant when the percentage difference in methylation was greater than 25% and a q value < 0.01. Differential methylation analysis and the DMCs annotations were performed with Methylkit and annotatr, two R/Bioconductor packages [77, 78], with the mm10 refGene and CpG islands were annotated from UCSC [79]. The annotation consisted of two categories: (i) regional annotation and (ii) distance to a CpG island. The distance annotation classified DMCs and DMRs according to whether they straddled a known CpG island, 2000 bp from regions flanking CpG islands (shores), 2000 bp from regions bordering the shores (shelves) or outside regions (open seas). The regional annotation consisted of classifying DMCs in four groups: intergenic regions, promoters, exons and introns.

**Table 1:** Main sequencing statistics

Sample name	Total reads	Uniquely aligned	Mapping efficiency (%)	CpGs Detected	CpGs cov >10	Average Coverage	Conv. Rate Meth. Spike-In (%)	Conv. Rate Unm. Spike-In (%)
SD_sample1	56,841,446	39,552,587	69	2,452,747	1,830,137	38	1.90	99.69
SD_sample2	42,686,853	29,690,766	69	2,370,803	1,702,974	30	1.58	99.67
SD_sample3	50,590,321	35,102,481	69	2,409,838	1,777,440	34	1.67	99.76
SD BPS1.5_sample1	77,445,640	53,608,226	69	2,527,456	1,908,307	49	1.63	99.74
SD BPS1.5_sample2	70,027,685	47,984,369	68	2,259,789	1,769,524	48	1.79	99.66
SD BPS1.5_sample3	47,817,786	32,953,242	68	2,366,331	1,717,666	33	1.71	99.71

#### Bioinformatic analysis of genome-wide methylation

Raw data and bioinformatics analysis were provided by Diagenode (Cat# G02020000) including percentage of hyper and hypomethylated region (in the form of pie chart and heatmap), CpGs and DMRs genomic distribution, identification and clustering of genes with DMRs in promoter or exons.

### Overlap analysis between transcriptome and genome-wide methylation data

For genes with an existing association between significant DNA hypo- or hypermethylation and mRNA up- or downregulation, a Pearson’s correlation test was performed by distinguishing DMRs in exon, promoter and intron. Likewise, an ontology analysis was performed using String database v 11.0 (https://string-db.org/).

## Supplementary Information

**Additional file 1.** List of abbreviations (names of genes) in order of appearance.

**Additional file 2.** Functional Gene Set Enrichment Analysis identified by the databases of pathways KEGG (A) or Reactome (B) for down-regulated genes and overexpressed genes using the gene set of genes passed filter criteria (Fold Change ≤ − 1.5 or ≥ 1.5 a, d *p*-value < 0.05).

**Additional file 3.** Gene Set Enrichment Analysis to highlight the network of transcription factor target triggered by BPS treatment using the gene set of genes passed filter criteria (Fold Change ≤ − 1.5 or ≥ 1.5 a, d *p*-value < 0.05).

**Additional file 4.** Gene Set Enrichment Analysis to highlight the alterations of gene functions triggered by BPS treatment.

**Additional file 5.** Gene Set Enrichment Analysis to highlight the network of transcription factor target triggered by BPS treatment.

**Additional file 6.** List of the connections between significant DNA methylation and gene expression deregulations induced by BPS treatment.

**Additional file 7.** Table reporting genomic distribution of differentially methylated CpGs and DMRs.

**Additional file 8.** List of genes of the heatmap in Fig. 1, in order of appearance from top to bottom.

**Additional file 9.** Functional Gene Set Enrichment Analysis identified by the databases of pathways KEGG or Reactome for differentially methylated cytosines in exons and/or promoters from liver DNA of C57Bl/6 J male mice exposed to BPS compare to control mice.

**Additional file 10.** Complete list of mRNA differential expression before application of fold threshold in TAC console.

**Additional file 11.** Complete list of DMR in exons and promoters after RRBS analysis.
