# Supplementary material for: Hepatic transcriptome and DNA methylation patterns following perinatal and chronic BPS exposure in male mice
Source: BMC Genomics. 2020 Dec 9;21:881. doi: 10.1186/s12864-020-07294-3 (PMC7727143; doi:10.1186/s12864-020-07294-3)
Supplement: Supplementary file 1 — Additional file 1. List of abbreviations (names of genes) in order of appearance. [file 12864_2020_7294_MOESM1_ESM.docx]

Additional file 1: List of abbreviations (names of genes) in order of appearance

Lipg : Lipase, Gys2 : Glycogen synthase 2, Elovl6 : ELOVL family member 6, Rorc : RAR-related orphan receptor gamma, Per2 : Period circadian clock 2, Mfsd2a : Major facilitator superfamily domain containing 2A, Fabp5 : Fatty acid binding protein 5, Lipe : Lipase hormone sensitive, Pcyt1a : Phosphate cytidylyltransferase 1, choline, alpha isoform, Thrsp : Thyroid hormone responsive, Lpin1 : Lipin 1, Fasn : Fatty acid synthase, Apoa4 : Apolipoprotein A-IV, Cyp2a4 : Cytochrome P450 family 2 subfamily a polypeptide 4, Cyp7a1 : Cytochrome P450 family 7 subfamily a polypeptide 1, Pfkfb1 : 6-phosphofructo-2-kinase/fructose-2,6-biphosphatase 1, Pck1 : Phosphoenolpyruvate carboxykinase 1, Gyg : Glycogenin, Fktn : Fukutin, Etnk2 : Thanolamine kinase 2, Hmgcr : 3-hydroxy-3-methylglutaryl-Coenzyme A reductase, Ces2b : Carboxyesterase 2B, Ces2c : Carboxyesterase 2C, Car5a : Carbonic anhydrase 5a, Dhcr7 : 7-dehydrocholesterol reductase, Chpf : Chondroitin polymerizing factor, Gpt2 : Glutamic pyruvate transaminase (alanine aminotransferase) 2, Tat : Tyrosine aminotransferase, Sds : Serine dehydratase, Hnmt : Histamine N-methyltransferase, Ido2 : Indoleamine 2,3-dioxygenase 2, Bhmt2 : Betaine-homocysteine methyltransferase 2, Tyrp1 : Tyrosinase-related protein 1, Acmsd : Amino carboxymuconate semialdehyde decarboxylase, Dhtkd1 : Dehydrogenase E1 and transketolase domain containing 1, Por : P450 (cytochrome) oxidoreductase, Abcb1b : ATP-binding cassette sub-family B (MDR/TAP) member 1B, Rdh11 : Retinol dehydrogenase 11, Car1 : Carbonic anhydrase 1, Nags : N-acetylglutamate synthase, Asl : Argininosuccinate lyase, Srd5a1 : Steroid 5 alpha-reductase 1, Cyp17a1 : Cytochrome P450 family 17 subfamily a polypeptide 1, Upp2 : Uridine phosphorylase 2, Dck : Deoxycytidine kinase, Pdxk : Pyridoxal (pyridoxine, vitamin B6) kinase, Coq10b : Coenzyme Q10 homolog B, Pdk2 : Pyruvate dehydrogenase kinase isoenzyme 2, Oxct1 : 3-oxoacid CoA transferase 1, Bhlhe41 : Basic helix-loop-helix family, member e41, Ecsit : ECSIT homolog, Gpam : Glycerol-3-phosphate acyltransferase, Slc25a11 : Solute carrier family 25 member 11, Per1 : Period circadian clock 1, Per3 : Period circadian clock 3, Gnao1 : Guanine nucleotide binding protein alpha O, Atp6v1c1 : ATPase H+ transporting lysosomal V1 subunit C1, Azin1 : Antizyme inhibitor 1, Camk2b : Calcium/calmodulin-dependent protein kinase II beta, Polr2g : Polymerase (RNA) II (DNA directed) polypeptide G, Gng2 : Guanine nucleotide binding protein (G protein) gamma 2, St3gal5 : ST3 beta-galactoside alpha-2,3-sialyltransferase 5, Alpl : Alkaline phosphatase, Gne : Glucosamine (UDP-N-acetyl)-2-epimerase/N-acetylmannosamine kinase, Usp2 : Ubiquitin specific peptidase 2, Noct : Nocturnin, Ciart : Circadian associated repressor of transcription, Slc20a1 : Solute carrier family 20 member 1, Egr1 : Early growth response 1, Dbp : D site albumin promoter binding protein, Arntl2 : Aryl hydrocarbon receptor nuclear translocator-like 2, Clock : Circadian locomotor output cycles kaput, Chchd7 : Coiled-coil-helix-coiled-coil-helix domain containing 7, Coq4 : Coenzyme Q4 homolog, Clpx : Caseinolytic mitochondrial matrix peptidase chaperone subunit, Slc30a10 : Solute carrier family 30 member 10, Agk : Acylglycerol kinase, Trim37 : Tripartite motif-containing 37, Tet1 : Tet methylcytosine dioxygenase 1, Foxa1 : Forkhead box A1, Foxa2 : Forkhead box A2, Arid5b : AT rich interactive domain 5B, Safb : Scaffold attachment factor B, Nudt5 : Nudix (nucleoside diphosphate linked moiety X)-type motif 5, Ivns1abp : Influenza virus NS1A binding protein, Fnip2 : Folliculin interacting protein 2, Insig1 : Insulin induced gene 1, Insig2 : Insulin induced gene 2, Elovl3 : ELOVL family member 3, Cyp2a5 : Cytochrome P450 family 2 subfamily a polypeptide 5, Ldlr : Low density lipoprotein receptor, Saa1 : Serum amyloid A 1, Srebf1 : Sterol regulatory element binding transcription factor 1, Mtf1 : Metal response element binding transcription factor 1, Irs1 : Insulin receptor substrate 1, Onecut1 : One cut domain, family member 1, Fgfr4 : Fibroblast growth factor receptor 4, Pitpnc1 : Phosphatidylinositol transfer protein cytoplasmic 1, Fmo5 : Flavin containing monooxygenase 5, Manea : Mannosidase endo-alpha, Pim3 : Proviral integration site 3, Bcl6 : B cell leukemia/lymphoma 6, Oit3 : Oncoprotein induced transcript 3, Mlst8 : MTOR associated protein, LST8 homolog, Glt1d1 : Glycosyltransferase 1 domain containing 1, Lcn2 : Lipocalin 2, Cish : Cytokine inducible SH2-containing protein, Map4k4 : Mitogen-activated protein kinase 4, Tnfaip2 : Tumor necrosis factor alpha-induced protein 2, Mt2 : Metallothionein 2, Pyroxd1 : Pyridine nucleotide-disulphide oxidoreductase domain 1, Cpeb4 : Cytoplasmic polyadenylation element binding protein 4, Klb : Klotho beta, Klhl12 : Kelch-like 12, Slc51b : Solute carrier family 51 beta subunit, Fam102a : Family with sequence similarity 102 member A, Nr0b2 : Nuclear receptor subfamily 0 group B member 2, Esrrg : Estrogen-related receptor gamma.
