# Supplementary material for: Hepatic transcriptome and DNA methylation patterns following perinatal and chronic BPS exposure in male mice
Source: BMC Genomics. 2020 Dec 9;21:881. doi: 10.1186/s12864-020-07294-3 (PMC7727143; doi:10.1186/s12864-020-07294-3)
Supplement: Supplementary file 3 — Additional file 3. Gene Set Enrichment Analysis to highlight the network of transcription factor target triggered by BPS treatment using the gene set of genes passed filter criteria (Fold Change ≤ − 1.5 or ≥ 1.5 a, d p-value < 0.05). [file 12864_2020_7294_MOESM3_ESM.docx]

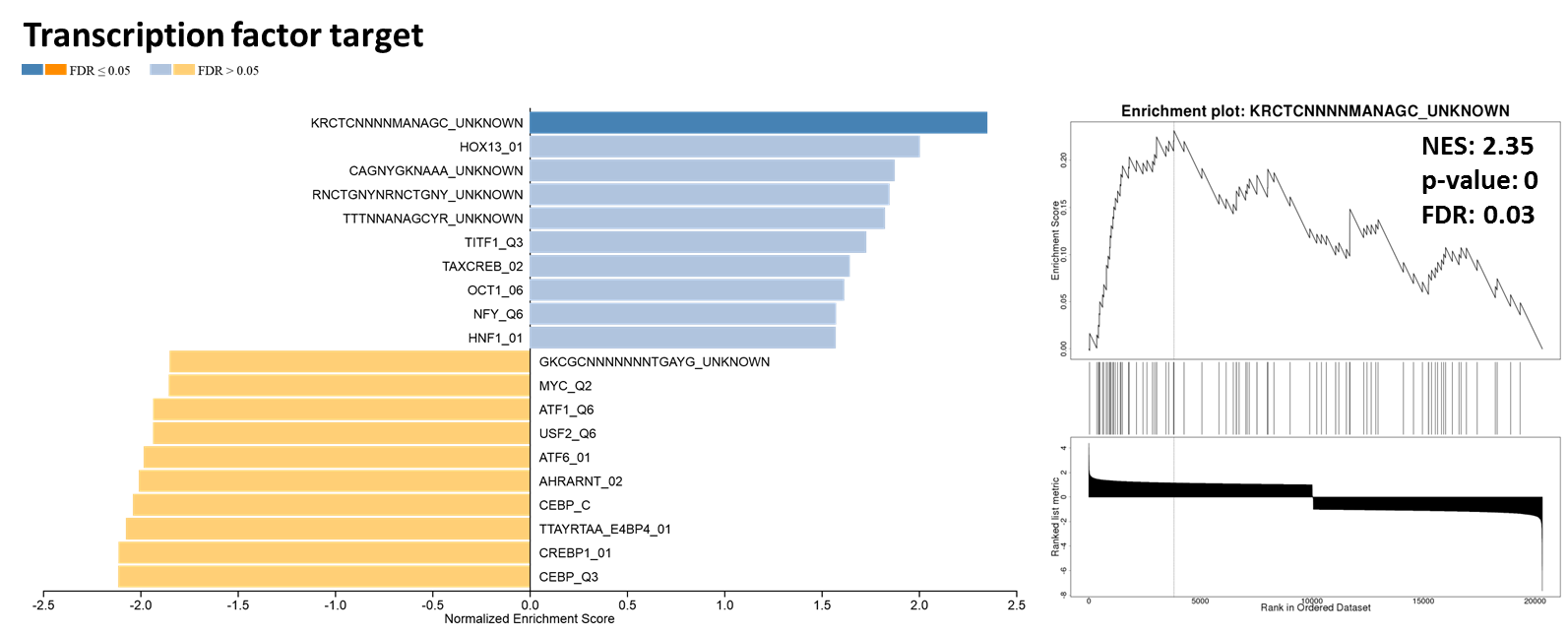


Additional file 3: Gene Set Enrichment Analysis to highlight the network of transcription factor target triggered by BPS treatment using the gene set of genes passed filter criteria (Fold Change ≤ -1.5 or ≥ 1.5 a,d p-value < 0.05) when we compare liver mRNA of C57Bl/6J male mice exposed to BPS from GD0 to 23 weeks-old at 1.5 µg/kg body weight/day with liver DNA of control mice by microarray. n = 3 pools of 3 animals each and by group.
