# Supplementary material for: Hepatic transcriptome and DNA methylation patterns following perinatal and chronic BPS exposure in male mice
Source: BMC Genomics. 2020 Dec 9;21:881. doi: 10.1186/s12864-020-07294-3 (PMC7727143; doi:10.1186/s12864-020-07294-3)
Supplement: Supplementary file 9 — Additional file 9. Functional Gene Set Enrichment Analysis identified by the databases of pathways KEGG or Reactome for differentially methylated cytosines in exons and/or promoters from liver DNA of C57Bl/6 J male mice exposed to BPS compare to control mice. [file 12864_2020_7294_MOESM9_ESM.docx]

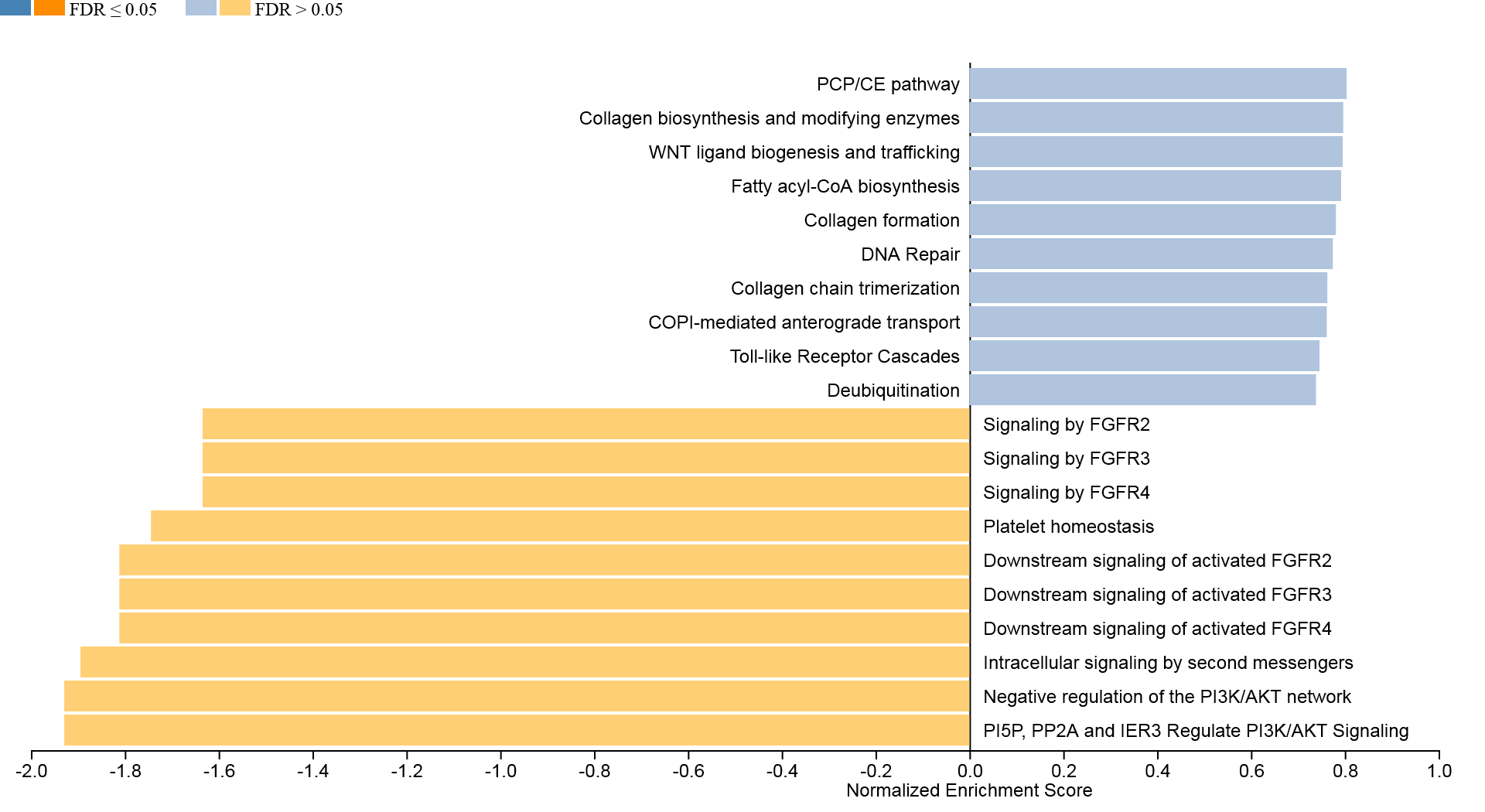

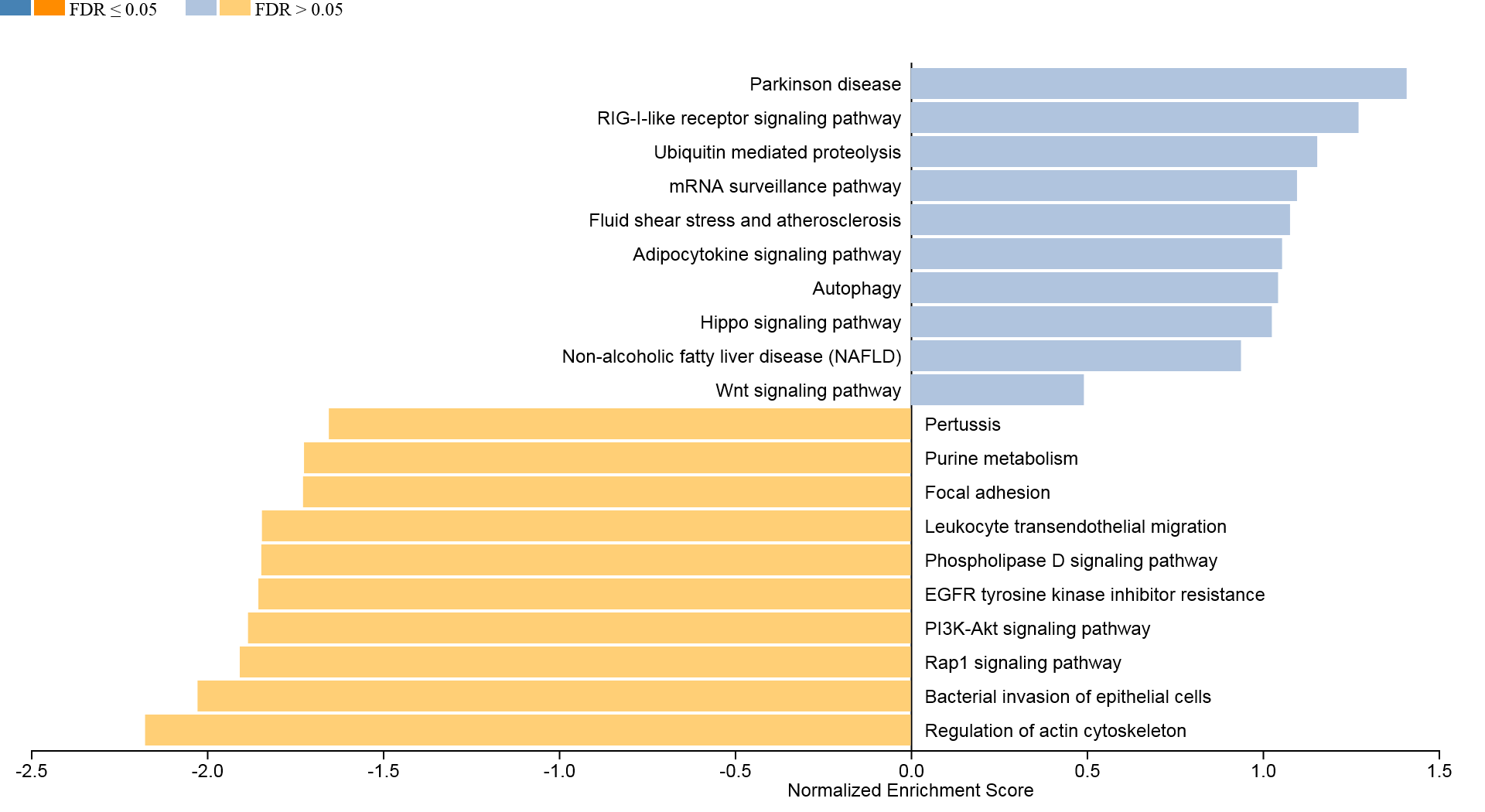


**Pathway Reactome**

**Pathway KEGG**

Additional file 9A: Functional Gene Set Enrichment Analysis identified by the databases of pathways KEGG or Reactome for differentially methylated cytosines in exons from liver DNA of C57Bl/6J male mice exposed to BPS from GD0 to 23 weeks-old at 1.5 µg/kg b.w./day is compared with liver DNA of control mice. n = 3 pools of 3 animals each and by group.


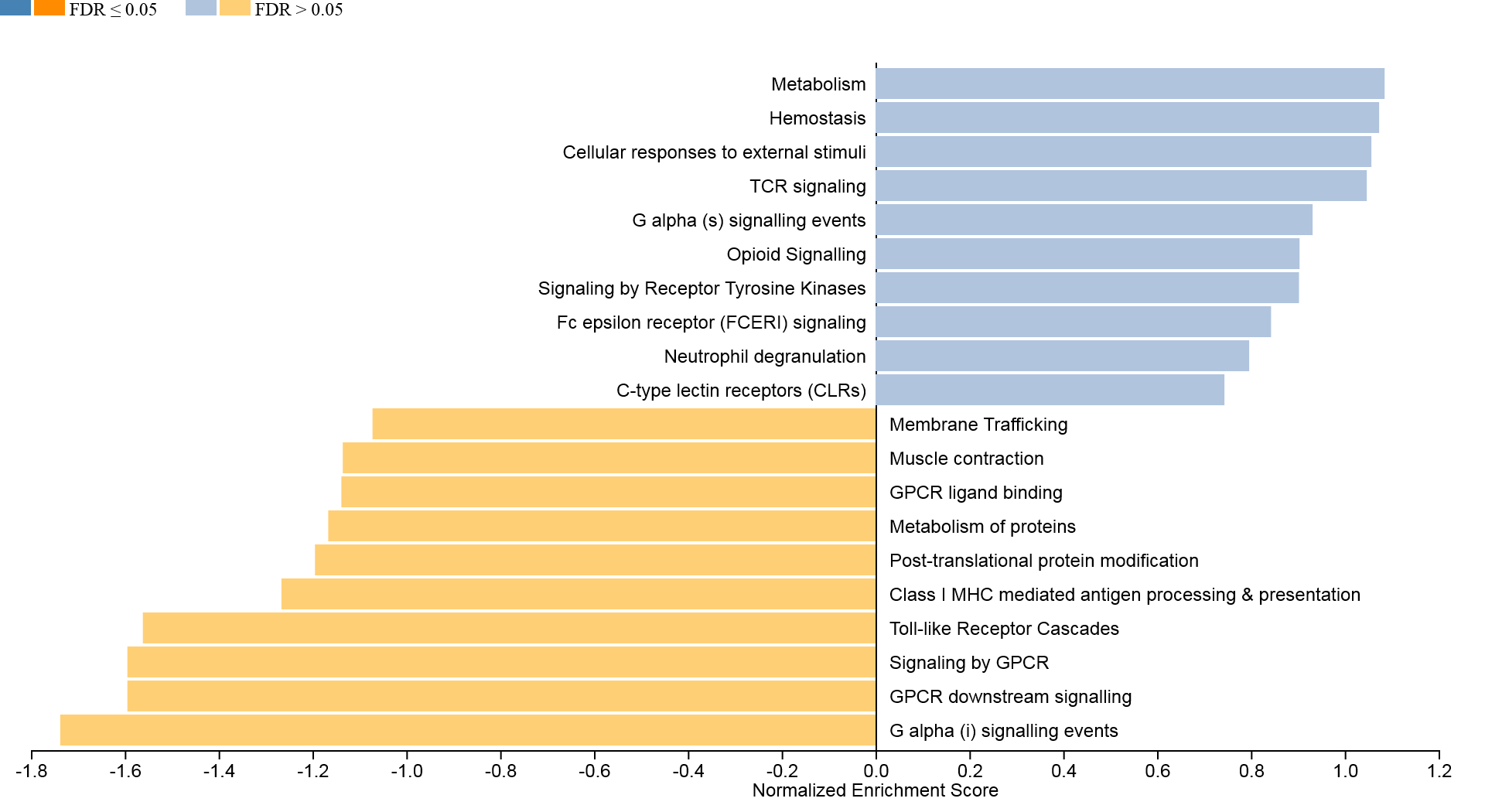

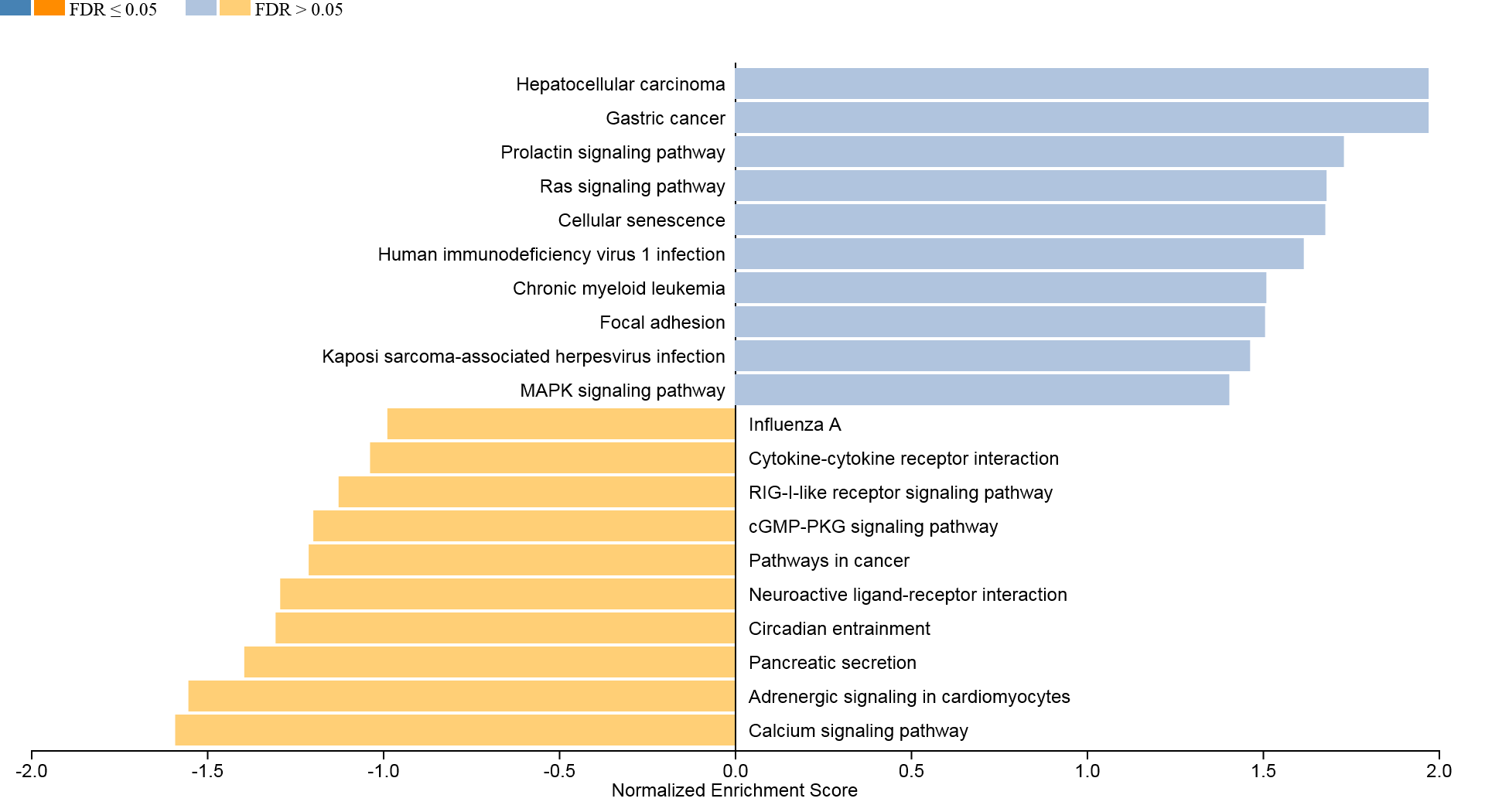


**Pathway KEGG**

**Pathway Reactome**

Additional file 9B: Functional Gene Set Enrichment Analysis identified by the databases of pathways KEGG or Reactome for differentially methylated cytosines in promoters from liver DNA of C57Bl/6J male mice exposed to BPS from GD0 to 23 weeks-old at 1.5 µg/kg b.w./day is compared with liver DNA of control mice. n = 3 pools of 3 animals each and by group.


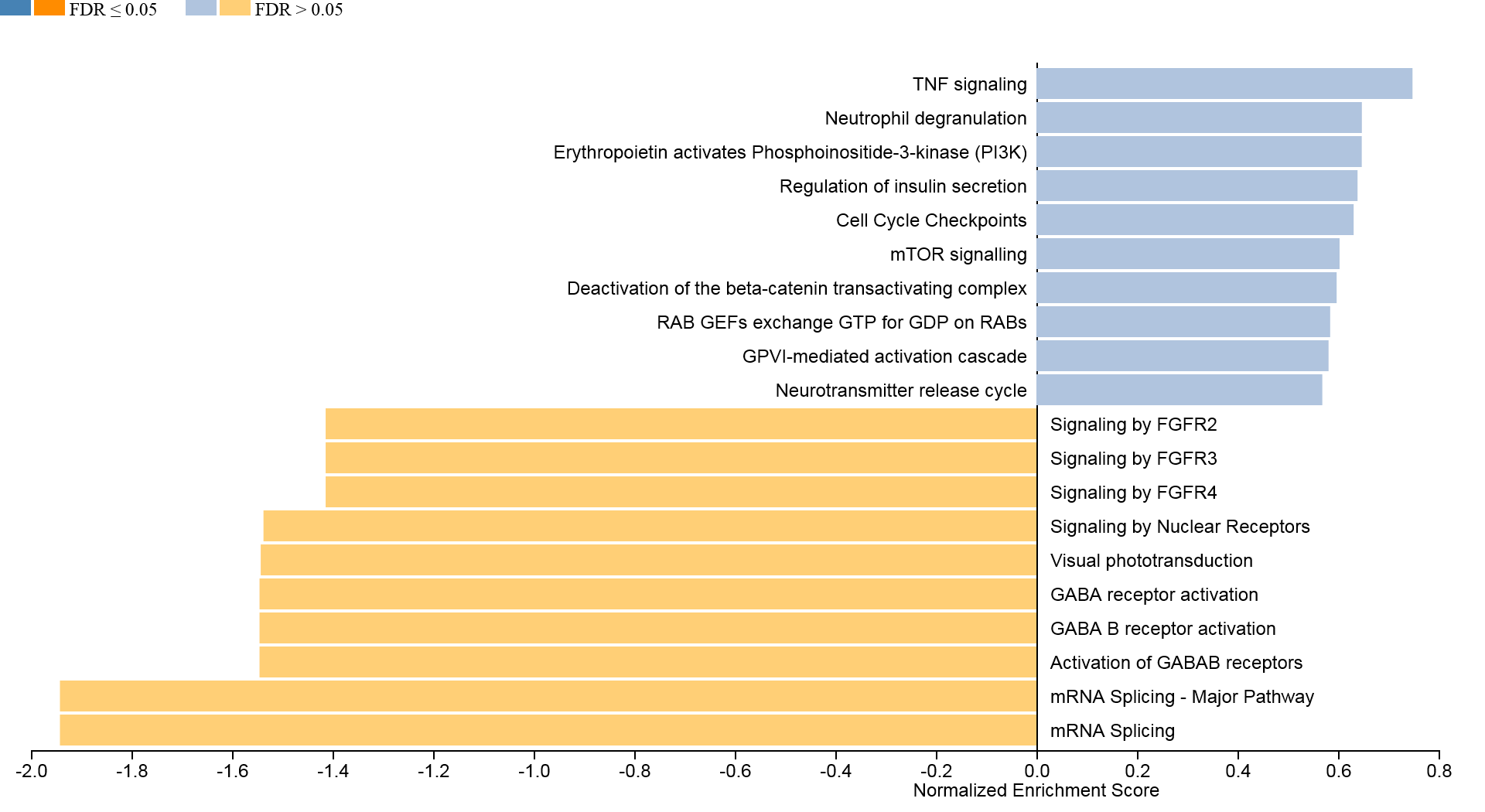

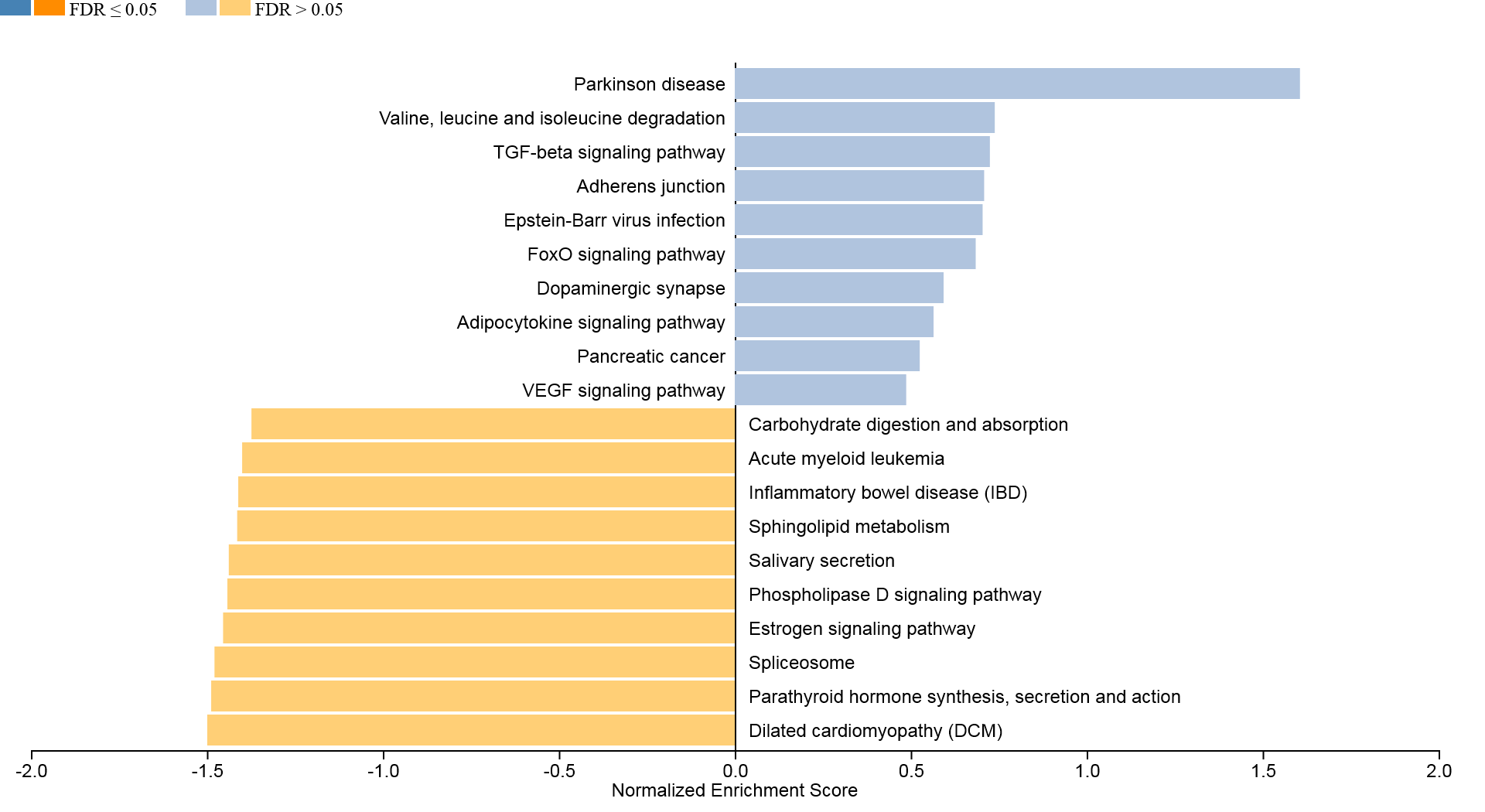


**Pathway Reactome**

**Pathway KEGG**

Additional file 9C: Functional Gene Set Enrichment Analysis identified by the databases of pathways KEGG (A) or Reactome (B) for differentially methylated cytosines in both exons and promoters from liver DNA of C57Bl/6J male mice exposed to BPS from GD0 to 23 weeks-old at 1.5 µg/kg b.w./day is compared with liver DNA of control mice. n = 3 pools of 3 animals each and by group.
